# Measuring transparency in the social sciences: political science and international relations

**DOI:** 10.1098/rsos.240313

**Published:** 2024-07-03

**Authors:** Bermond Scoggins, Matthew P. Robertson

**Affiliations:** ^1^ School of Politics and International Relations, Australian National University, Canberra, Australia; ^2^ School of Social Sciences, Monash University, Melbourne, Australia; ^3^ Department of Social Data Science, University of Mannheim, Mannheim, Germany

**Keywords:** data sharing, preregistration, open science, journal policy

## Abstract

The scientific method is predicated on transparency—yet the pace at which transparent research practices are being adopted by the scientific community is slow. The replication crisis in psychology showed that published findings employing statistical inference are threatened by undetected errors, data manipulation and data falsification. To mitigate these problems and bolster research credibility, open data and preregistration practices have gained traction in the natural and social sciences. However, the extent of their adoption in different disciplines is unknown. We introduce computational procedures to identify the transparency of a research field using large-scale text analysis and machine learning classifiers. Using political science and international relations as an illustrative case, we examine 93 931 articles across the top 160 political science and international relations journals between 2010 and 2021. We find that approximately 21% of all statistical inference papers have open data and 5% of all experiments are preregistered. Despite this shortfall, the example of leading journals in the field shows that change is feasible and can be effected quickly.

## 1. Introduction

The Royal Society has as its motto the injunction *Nullius in verba*: ‘Take nobody’s word for it’. Yet a large portion of published studies in the social sciences demand of the reader exactly this.

Over the past several decades, open science advocates have called for the routinization of open science practices such as posting data and code upon a paper’s publication and the preregistration of experiments [1]. Beginning principally in the psychological sciences, advocacy for these reforms rose in the 2010s due to large-scale replication failures of prominent psychological studies which highlighted the widespread presence of false-positive findings [2,3].

Open science practices bolster the credibility of a field and its findings by allowing the readers to evaluate the methods by which researchers reach their conclusions. While trust is the currency of every epistemic community, the demand for trust alone weakens credibility. If data and code are available, interested researchers can ensure a finding’s results are computationally reproducible, robust to alternative model specifications and error free. For experiments (i.e. randomized controlled trials), preregistration allows the reader to determine whether there was a selective exclusion of hypotheses, measurements or statistical analyses that run counter to the author’s favoured hypotheses.

Concern for research transparency has become more salient over the past decade as scholars recognize that the accumulation of false positives can drive unsuccessful decision-making and interventions. This leads to inefficient resource allocation and weakens the credibility of a field. In fields like medicine, open science practices have been strongly advocated in recognition of the direct harm that false positives can cause [4,5]. Leading journals in political science and international relations are increasingly mandating the provision of data and code, as well as encouraging the preregistration of experiments.

We distinguish *computational reproducibility*—making available the data and code of a paper’s results, for others to reproduce them—from *replicability*—where new data are collected using an identical or conceptually similar design to the original paper [6,7]. Usage of these terms has been inconsistent between fields. Political science, unlike psychology, conducts fewer experimental studies and what is often termed ‘replication’ is actually about computational reproducibility [1,8].

Political science and international relations appear to have taken open science practices seriously, with high-profile journals and academics endorsing initiatives like the Data Access and Research Transparency (DA-RT) statement. This has led some scholars to believe that the problem of open data has mostly been solved. Yet current assessments of the field’s progress have been based on relatively small samples and time-intensive human coding procedures [9–12].^1^

Our paper presents the largest-scale study of open science practices in political science and international relations thus far; it is also the first systematic study of the prevalence of preregistration in experiments in these fields. Our study spans the years 2010–2021 and includes population-level data, allowing us to illustrate trends in specific journals. Documenting such trends is important given the key role played by journals in promulgating and enforcing transparent research norms.

We ask two questions: (i) What proportion of papers that rely on statistical inference make their data and code public? and (ii) What proportion of experimental studies were preregistered? We gather 93 931 published articles from the top 160 journals ranked by Clarivate’s Journal Citation Reports [13] and use machine learning classifiers to identify either statistical inference or experimental papers.^2^ We identified which had open data and preregistration using public application programming interfaces (APIs), text analysis and web scraping.

### 1.1. The state of open political science practices

Since the onset of the replication crisis, how much of the literature dependent on data and statistical inference still relies solely on reader trust? Extant research on the prevalence of open data practices in political science paints a sobering picture. Stockemer *et al.*’s [10] analysis of 145 quantitative studies published in three journals during 2015 found that only 55% provided original data and 56% provided code.^3^ An earlier analysis, conducted on 494 quantitative articles in six leading political science journals between 2013 and 2014, found that full computational reproducibility materials (data and code) were available for only 58% of papers [9].^4^

Poor data availability affects many natural and social science disciplines [14,15]. A random sample of 250 psychology papers published between 2014 and 2017 estimated that 14% of papers shared research materials, 2% provided original data and 1% shared their code [16]. Preregistration was rare (3%). Similarly, even once data are shared, analytic reproducibility is not guaranteed [17].

A tonic for many of these problems is straightforward: computational reproducibility materials for all quantitative studies and preregistration for experiments. Reproducibility materials and preregistration militate against questionable research practices (QRPs) that lead to false positives by constraining researcher degrees of freedom and ensuring that key decisions made in the analysis process are transparent to peers.

In the behavioural sciences, false positives can arise from decisions that are rationalized as legitimate by authors: failing to report all dependent variables in a study, collecting more data after seeing whether the results were statistically significant, failing to report all conditions, stopping data collection after achieving the desired result, rounding down *p* values, selectively reporting studies that ‘worked’, selectively excluding observations and claiming an unexpected finding was predicted (or hypothesizing after results are known). However, these practices obfuscate the uncertainty around a particular set of claims and mislead readers into being overconfident about a study’s conclusions.

The use of QRPs appears to be widespread in many of the social sciences. Surveys of psychology and criminology researchers report they routinely do not report all dependent variables, collect more data after peeking at results and selectively report statistically significant studies [18,19]. Other methods of detecting publication bias, such as analysing sets of studies or literatures using a *p*-curve or *z*-curve, reveal extensive clustering of *p* values (*z* scores) just past *p* < 0.05 [20,21]. Examples of these problems in the behavioural and social sciences range from the power-posing literature [22] to economic research using instrumental variables and difference-in-differences [23].

In recognition of these problems, professional organizations in political science and international relations, including the American Political Science Association (APSA), have led efforts to increase the availability of data and code that accompany published papers. The DA-RT statement developed by the APSA council in 2014 involved a commitment by journal editor signatories to increase the availability of data ‘at the time of publication through a trusted digital repository’, as well as require authors to ‘delineate clearly the analytic procedures upon which their published claims rely, and where possible to provide access to all relevant analytic materials’ [24].

While there was an intramural debate about how DA-RT standards would affect qualitative work, given the heterogeneity of interview data and other forms of qualitative analysis, we bypass these arguments in this article by focusing exclusively on papers relying on statistical inference.^5^ It is relatively straightforward for researchers using statistical inference to release the very data and code that were necessary to produce the results in their papers. As Key [9] notes, the Internet has reduced the cost for journals to set up Dataverse repositories and made it easier for researchers to share their data and code. Rising usage of free statistical programming software, such as R and its desktop application RStudio, also reduces barriers to computational reproducibility.

The 27 journal editors who adopted the statement agreed to implement reforms by January 2016. Of the 16 DA-RT signatory journals in our dataset, two made no change in practice and a further four have data and code that is difficult to accurately estimate.^6^

### 1.2. The need for open data

#### 1.2.1. Uncovering data errors and misinterpretation

Errors in data or the misreporting of *p* values or test statistics inevitably occur in research and can go undetected by an article’s authors or peer reviewers. These problems, if addressed, may substantively alter an article’s conclusions or produce null rather than positive results. Reporting errors in regression coefficients or test statistics occur frequently [27].

Access to the original data can help determine whether errors are trivial, and contribute to retraction efforts if they are not [28]. In some cases, access to the data allows for detailed concerns with a paper’s analysis to be illustrated without the journal believing a retraction is warranted [29,30].

#### 1.2.2. Identifying model misspecification and facilitating extension

Researchers have tremendous flexibility in deciding how to collect data and which statistical models should be specified to analyse them. Gelman & Loken termed this process the ‘garden of the forking paths’ [31]: some set of decisions might yield a positive result, while another set of equally justifiable decisions might lead to a null result. The replication crisis has shown that it is a mistake to view a single study or set of statistical analyses as a definitive answer to a given theory or claim—the scientific process should instead be iterative, exploratory and cumulative [32].

Open data can address the problem of model misspecification and uncertainty around modelling the data-generating process [33]. Since researchers cannot anticipate changes to methodological best practices, computational reproducibility materials allow scholars to make adjustments if best practices change.^7^ Even if misspecification is not a problem, extending and building off of the original analyses—to run more theoretically motivated models, sensitivity analyses or assess treatment heterogeneity—are net positives for science [35].

#### 1.2.3. Exposing data falsification

In the most egregious cases, open data allow researchers to investigate and expose data falsification. High-profile exposures of data falsification include the LaCour & Green [36] case in political science and the Shu *et al*. [37] case in psychology [38,39]. Both rested on investigator access to the original data. While presumably data falsification is exceedingly rare, there is no way to know its extent given the general absence of computational reproducibility materials in the first place.

### 1.3. The need for preregistration

#### 1.3.1. Distinguishing confirmatory from exploratory analysis

Preregistration means that researchers specify their hypotheses, measurements and analytic plans prior to running an experiment. This commits researchers to making theoretical predictions before they can view the data and be influenced by observing the outcomes [2,40]. By temporally separating predictions from the data that test their accuracy, there is much less flexibility for both *post hoc* theorizing and alterations of statistical tests to fit the prediction.

*Post hoc* theorizing, also known as hypothesizing after the results are known (HARKing), is an example of circular logic—the researcher conducts many tests when exploring a dataset, the data reveal a relationship that can be made into a hypothesis, and that hypothesis is ‘tested’ on the data that generated it [41]. But the diagnosticity of a *p*-value is in part predicated on knowing how many tests were performed: when an exploratory finding is reported as a prediction, the normal methods employed to evaluate the validity of a hypothesis—such as whether the *p*-value is <0.05 (i.e. null hypothesis significance testing)—no longer hold. The *p* values in that case have unknown diagnosticity [41]. Thus, *post hoc* theorizing and selective reporting greatly contribute to false positives.

#### 1.3.2. Reducing the selective reporting of results

The selective reporting of statistical tests and results can occur for a variety of reasons. There are numerous legitimate ways of analysing data, and this makes selective reporting seem justifiable. Danger arises when researchers convince themselves that the measures and tests lending evidence to their claims are the ‘right’ ones, while unjustifiably failing to report measures and tests that did not support the favoured hypothesis.

Selectively reported experimental studies often result in overconfident theoretical claims and inflated effect sizes when compared to replications. The Open Science Collaboration [3] and Many Labs studies [42,43] have shown that the effect sizes in highly powered replications are much smaller than those in the original studies. When reforms are implemented mandating preregistration, by research bodies or formats like registered reports, the number of null results reported rises [44,45].

The primary purpose of preregistration is to provide journal reviewers and readers the ability to transparently evaluate predictions and the degree of flexibility researchers had to arrive at their conclusions [46–48]. It is up to the reader to determine whether preregistered studies followed their preregistration plan and adequately justified deviations—insufficiently detailed preregistration reports are an ongoing problem [49].

The replication crisis has altered best practices and changed the habits of many researchers in the behavioural sciences. As we show below, preregistration is not yet the norm in political science and international relations. The conclusions from many studies relying on statistical inference, even some that have been preregistered on a registry, remain exposed to the statistical pitfalls described above.

## 2. Methods

Our study design called for a comprehensive analysis of population-level data, yet our populations—(i) papers using data and statistics and (ii) original experiments—were embedded in a larger population of *all* political science and international relations publications in target journals. We downloaded all of the journals’ papers from 2010 to 2021. Once we had these papers locally, we identified the data, statistical and experimental papers through dictionary-based feature engineering and machine learning. We then used public APIs, Web scraping and text analysis to identify which of the studies had computational reproducibility materials. We outline this process below.

### 2.1. Phase 1: gathering and classifying the papers

We used Clarivate’s 2021 Journal Citation Report to identify target journals. We filtered for the top 100 journals in both political science and international relations, and combined the two lists for a total of 176 journals.^8^

With this list, we used the Crossref API to download all publication metadata. We were able to obtain records for 162 journals. This resulted in over 445 000 papers, which we then filtered on Crossref’s published.print field for 2010 and onwards, resulting in 109 553 papers. We used the published.print field as it was the only reliable indicator of the actual publication date, and the most complete.^9^ As of mid-2023, we were able to obtain 93 931 of these articles in PDF and HTML formats, and we use this as the denominator in the study.

We converted the PDFs to plaintext using the open-source command line utility pdftotext, and we converted the HTML files to text using the html2text utility.^10^

Identifying the papers that relied on data, statistical analysis and experiments was an iterative process. In each case, we read target papers and devised a dictionary of terms meant to uniquely identify others like them. We iteratively revised these dictionaries to arrive at terms that seemed to maximize discrimination for target reports. The dictionaries eventually comprised 52, 180 and 133 strings, symbols or regular expressions for the three categories, respectively.^11^

The dictionaries were then used with custom functions to create document feature matrices (DFMs), where each paper is an observation, each column a dictionary term and each cell a count of that term.^12^ The DFM format made the papers amenable to large-scale analysis. In machine learning parlance, this process is known as feature engineering.

For the first research question—examining the presence of code and data in papers involving statistical inference—we hand-coded a total of 1624 papers with Boolean categories and identified 585 that relied on statistical inference. We defined statistical inference papers as any that involved mathematical modelling of data. These include terms associated with the specification of statistical models like ordinary least squares, regression and control groups or variables.^13^ This definition is meant to capture a simple idea: mathematical modelling requires computer instructions that perform functions on numbers. In the absence of computational reproducibility materials, these transformations cannot be exactly reproduced by readers. We also developed a dictionary of 35 terms for formal theory papers, because we wished to exclude papers that did not apply a model to real-world data.

For the second question—examining what proportion of experiments were preregistered—we hand-coded 518 papers with a single Boolean category: whether the paper reported one or more original experiments. We defined this as any article containing an experiment where the researchers had control over treatment.

We then trained two machine learning models—the support vector machine (SVM) and naive Bayes (NB) binary classifiers—to arrive at estimates for the total number of statistical inference and experimental papers.^14^ SVMs are pattern recognition algorithms that give binary classifications to variables in high-dimensional feature space by finding the optimal separating boundary between labelled training data [50,51]. The NB family of algorithms calculate the posterior probability of a given classified input based on the independent probability of all the values of its features; it then applies this trained algorithm to classify new inputs [52].

We report the SVM model results both for their greater accuracy and due to our theoretical prior that the model would be more suitable for a high-dimensional classification problem. For the first research question, our SVM model achieved 92.35% accuracy for statistical papers. For the classifying experiments, the accuracy was 86.05%. In appendix A, we report the confusion matrices, hyperparameter tuning data and NB models.

The application of the SVM model to the full dataset of 93 931 publications leads to an estimate of 24 026 using statistical inference.

The identification of experimental papers proceeded slightly differently. Rather than beginning with the full corpus, we first filtered for only the papers that included the word ‘experiment’ over five times (4835). We then ran the SVM classifier on this subset. The resulting estimate was 2552 papers reporting experiments.

### 2.2. Phase 2: identifying open data and preregistrations

We attempted to identify open data resources in seven ways:

— Using the Harvard Dataverse API, we downloaded all datasets held by all journals in our corpus who maintained their own, named Dataverse (*n* = 20).^15^
— We queried the Dataverse for the titles of each of the 109 553 papers in our corpus and linked them to their most probable match with the aid of a custom fuzzy string-matching algorithm. We validated these matches and manually established a string-similarity cut-off, setting aside the remainder.^16^
— We extracted from the full text of each paper in our corpus the link to its dataset on the Dataverse (1142; note this had significant overlap with the results of the first and second queries).^17^
— We downloaded the metadata listing the contents of these datasets, to confirm first that they had data in them, and second that they did not consist of only pdf or doc files. In cases where a list of metadata was not available via the Dataverse API, we scraped the html of the dataset entry and searched for text confirming the presence of data files.^18^
— We used regular expressions to extract from the full-text papers references to ‘replication data’, ‘replication materials’, ‘supplementary files’ and similar terms, then searched in the surrounding text for any corresponding URLs or mentions of author’s personal websites or other repositories.^19^ We validated these results by exporting various combinations of string matches with the above terms to Excel files, where we examined them in tabular format and validated their relevance. Given that replication and supplementary material stored on personal websites are not of the same order as material on the Dataverse and similar repositories, these results are recorded in appendix A under the rubric of ‘precarious data’.^20^
— We searched all of the full-text papers for references to other repositories, including Figshare, Dryad and Code Ocean, using regular expressions, where the surrounding text contained a URL fragment.^21^
— As additional validation for DA-RT signatory journals specifically, we downloaded the html file corresponding to each article and/or the html file hosting supplemental material (*n* = 2284), then extracted all code and data-related file extensions to establish their open data status.^22^

We attempted to identify preregistration of experiments in the following ways:

— We used regular expressions to extract from all of the experimental papers sentences that referred to ‘prereg’ or ‘pre-reg’, ‘preanalysis’ or ‘pre-analysis’, as well as any references to commonly used preregistration servers (OSF, EGAP and AsPredicted), and then searched for the availability of the corresponding link to validate that the preregistration had taken place. Parts of this process—for instance, searching author names in the Experiments in Governance and Politics (EGAP) registry to look for the corresponding paper—involved time-consuming detective work.^23^
— We downloaded all EGAP preregistration metadata in JSON format from the Open Science Framework Registry (https://osf.io/registries/), extracted from this file all osf.io links and unique EGAP registry IDs and used command line utilities to search for them through the corpus of all the papers.^24^

We did not examine whether the published report conformed to the preregistration plan.

## 3. Results

**Figure 1.**
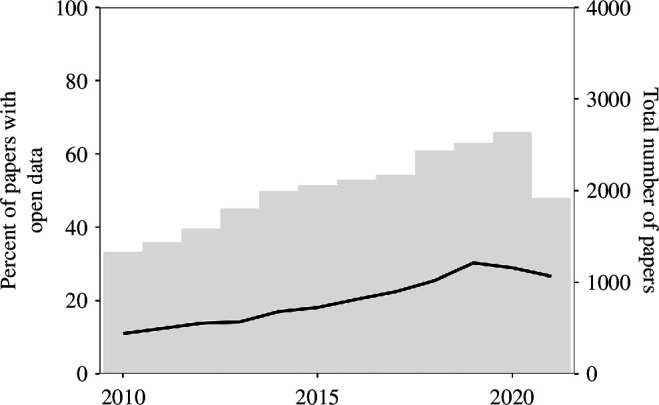
Open data in statistical inference papers by year.

Statistical inference papers are infrequently accompanied by the datasets or code that generated their findings. For the 12-year period under observation, we were able to match 21% of statistical inference articles to data repositories (overwhelmingly the Harvard Dataverse). Encouragingly, figure 1 shows that the percentage of open data has increased between 2010 and 2021—rising steadily from about 11% to 26% during this period.

The total number of statistical inference papers has gradually increased during the 12-year period. In 2010, we found 1329 papers and 2640 in 2020—the last year with complete data. This supports King’s [53] observation that political science and international relations have long been disciplines increasingly concerned with quantitative methods.^25^ While the percentage of papers with open data have increased, so too have the absolute number of statistical papers without it. There are simply more published papers making inferences based on hidden data.

**Figure 2.**
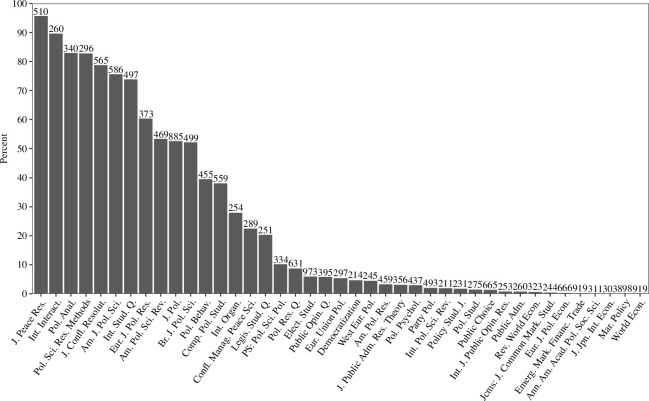
Open data in statistical inference papers by journal (with over 200 papers). The number of detected papers by journal are above each bar.

There are significant differences in open data practices between journals. Figure 2 displays the percentage of statistical inference papers with open data in the 41 journals with over 200 such papers.^26^ The number above each journal’s bin represents the number of statistical inference papers detected by the SVM classifier. Of the 41 journals displayed, 11 have over 50% open data and 16 have over 20%.^27^ The distribution of open data by journal in figure 2 shows the stark divide between the quarter of journals that have high open data rates and the three quarters which do not. In 2020, however, improvements had occurred. Of the 52 journals with over 20 statistical inferences, 16 had over 50% open data. A further 5 had over 20%.

**Figure 3.**
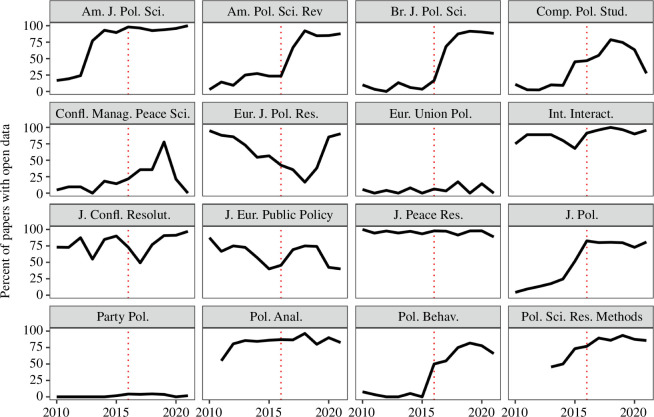
Open data in statistical inference papers by year published in 16 of the 27 journals signatory to the DA-RT statement.

The effectiveness of the DA-RT statement on journal open data practices is illustrated in figure 3, which displays the percentage of statistical inference papers with open data by year in each of the 16 DA-RT signatory journals we consider.^28^

Four journals*—American Journal of Political Science*, *International Interactions*, *Political Analysis* and *Political Science Research and Methods*—had already made significant progress prior to the release of the initial DA-RT guidelines in 2014. Many of the remaining journals either made significant progress in 2016 or shortly thereafter.

One caveat is that 2 of the 16 journal signatories have consistently low levels of open data even after DA-RT reforms were agreed to commence on 15 January 2016. The extent of transparent practices in three other journals—*Journal of European Public Policy*, *European Journal for Political Research* and *Conflict Management and Peace Science*—was more difficult to determine, given they did not use the Harvard Dataverse. Our attempt to estimate data and code availability for such journals, noted in point 7 of phase 2 of §2, appears to produce unreliable and puzzling results.

**Figure 4.**
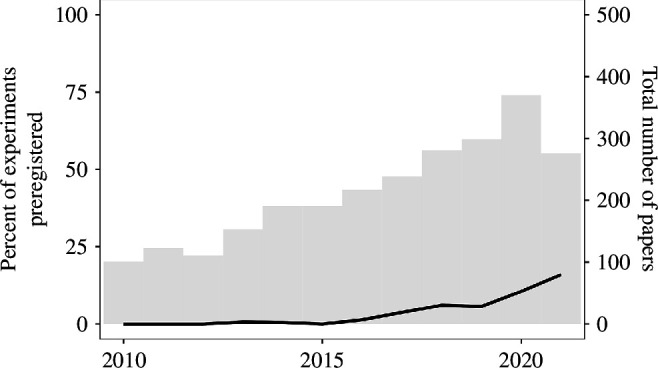
Preregistration in experiments by year.

The preregistration of experiments is rare in political science and international relations journals. Figure 4 shows that the first preregistered study in the dataset that we could identify was in 2013, and that the rate of preregistration only began climbing in 2016. The proportion of experiments that were preregistered for the entire period is approximately 5%; the annual rate has slowly risen to 16% in 2021.

**Figure 5.**
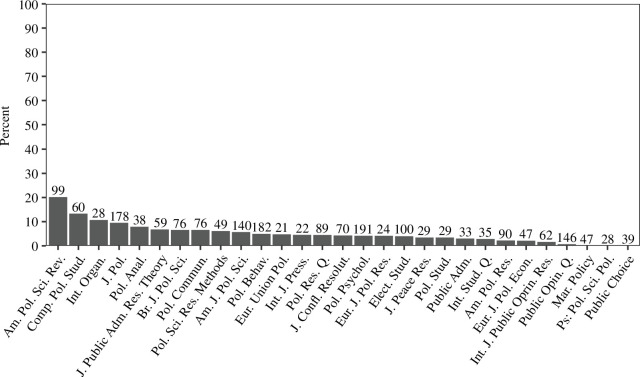
Preregistration in experiments by journal (with over 20 papers). The number of detected experiments by journal is above each bar.

Figure 5 shows the percentage of experiments that were preregistered in the 29 journals with more than 20 experiments. Only the *American Political Science Review* exceeds 20%. Unlike with open data, when it comes to preregistration the differences between journals are small. Of the experiments published in *Political Psychology* and *Political Behaviour*, the two journals with the most experiments that bridge the gap between political science and psychology, only 4% and 5%, respectively, are preregistered.

Prior to the onset of the replication crisis, beginning with psychology in the 2010s, there were no organized attempts at enforcing preregistration or using registered reports as a way of curbing researcher flexibility and its attendant QRPs. As psychology was among the first of the sciences to reckon with its methodological issues, brought to light in part by such articles as Simmons *et al.*’s [2], it is logical that it took several years for these new practices to be adopted in contiguous disciplines like political science and international relations. However, our data illustrate that significant improvements must be made in order for experiments in these fields to meet current methodological best practices.

## 4. Discussion

Scientists must carry out their work while simultaneously signalling and vouchsafing for its credibility. For the pioneers of the scientific method in seventeenth-century Europe, this included an ensemble of rhetorical and social practices, including the enlistment of trusted witnesses to testify that experiments had in fact taken place as claimed—this is what Shapin refers to as the moral economy of science [54,55].

In the digital age, we argue that the credibility of social science must largely rest on computational reproducibility. The same goes for preregistration and experiments. Adhering to these practices ensures other social scientists can check and reproduce the findings and demonstrates a commitment to the norm of science as a shared enterprise.

The chief reason for depositing code and data is not for signalling: open science practices provide the reader with an opportunity to transparently evaluate the evidence for a set of claims and scrutinize an article for any of the myriad problems that plague the use of data and statistical models. An interested reader could investigate an article’s data and code for errors, determine whether results are robust to different model specifications or, in rare cases, detect data falsification. For experiments, the published paper can be compared with the preregistration document to determine whether there were any unjustified deviations.

**Figure 11. F11:**
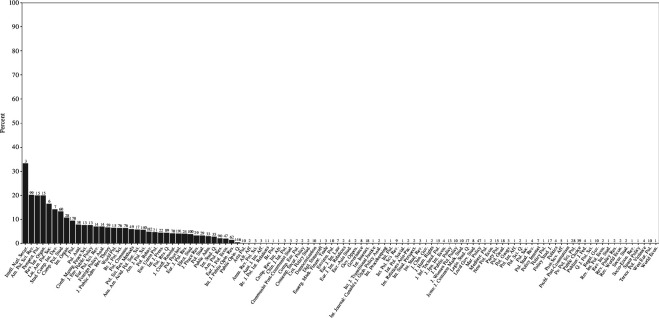
Preregistration by journal.

Our findings show that political science and international relations are not currently living up to these best practices. For the approximately 25 000 statistical inference papers in the dataset, we could only identify approximately 21% that had a corresponding data repository. Despite improvement in most years, change has not been uniform across the discipline—most of the progress has been made by a handful of the highest impact factor journals. In 2020, for example, 16 out of the 52 journals with over 20 statistical inference papers had an open data percentage over 50% (figure 11)—20 journals had an open data percentage over 20%. We could not locate data or code for 2 of the 16 DA-RT signatories in our dataset.

Universal open data is a collective action problem, and it is the responsibility of journals to foster and enforce these disciplinary norms. In the absence of that, individual researchers do not always share data and requesting it can sometimes be mistaken as a gesture of challenge rather than collegiality. As Simonsohn [56] notes, the modal response to his requests for original data was that the data were no longer available. We suspect that variation in open data practices between journals reflects differences in journal editors’ views of its importance for research credibility.

The DA-RT initiative sparked spirited debate in the field about the provision of data and code—but the same cannot be said for preregistration. Experiments are rarely preregistered. Of the roughly 2552 experiments in our dataset, 5% are preregistered. Given that the use of experiments only began to take off after 2015, as shown in figure 4, the proportion of preregistered experiments in the literature is understandably low. Fortunately, the trend is positive. One journal of 26 with more than 5 published experiments had a preregistration percentage of over 30% in 2020. While we do not take a position on whether all experiments should be preregistered, the percentages we identify should stimulate discussion on what the optimal percentage should be.

Identifying whether an experiment had a corresponding preregistration report was at times difficult. Numerous experiments made no mention of their preregistration report in the manuscript despite having one listed in a repository. Locating it was also difficult given the changing manuscript titles and authors. Their omission in the manuscript is probably owing to the fact that many journal editors do not determine whether an experiment has a preregistration or pre-analysis plan or request their disclosure.^29^

The difficulty of matching an experiment with its preregistration report is far smaller than matching a manuscript to a concealed preregistration report. A unique and unanticipated problem we found was the authors publishing a study where they omitted any reference to a preregistered experiment—ostensibly due to null findings. Byun *et al.* [57] use their survey data to make descriptive claims while failing to discuss the design or results of their experimental manipulation [58]. It is not clear whether their results failed to further their own argument or were possibly disconfirmatory. In either situation, readers are not permitted to transparently evaluate the strength of their claims.

Peer reviewers and readers of published works routinely examine whether a theory or explanation has appropriate evidence; whether the measurements are valid and reliable; whether the model has been appropriately specified. Here, we prompt referees and readers to also begin asking: (i) Are the computational reproducibility materials on the Harvard Dataverse or some other reliable repository? (ii) Is the paper computationally reproducible based on those materials? (iii) If an experiment, was it preregistered? (iv) Does the analysis in the experimental paper follow the preregistration plan and are deviations from that plan justified?^30^ We hope that evaluating scientific research in this manner will help move readers away from trusting research in the absence of open science practices to a more informed trust in their presence.

## Figures and Tables

**Figure 6.**
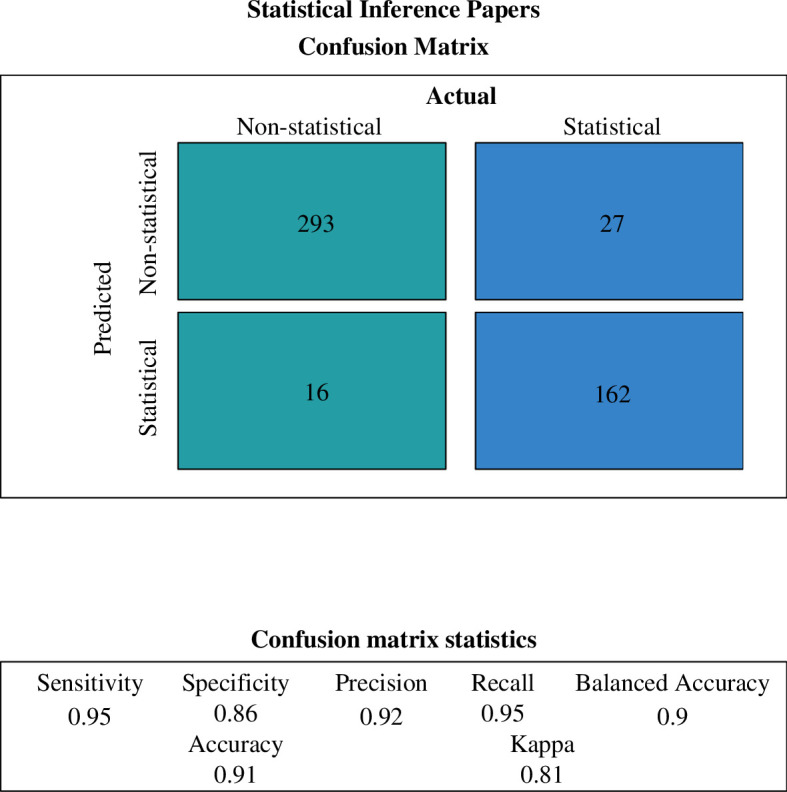
SVM confusion matrix for statistical inference paper classification.

**Figure 7. F7:**
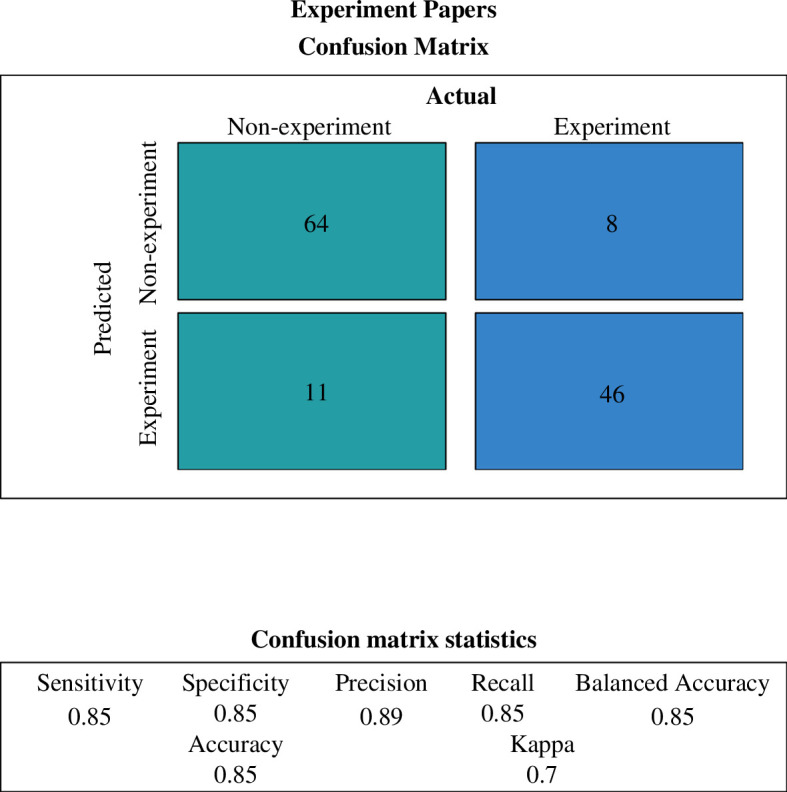
SVM confusion matrix for experiment paper classification.

**Figure 8. F8:**
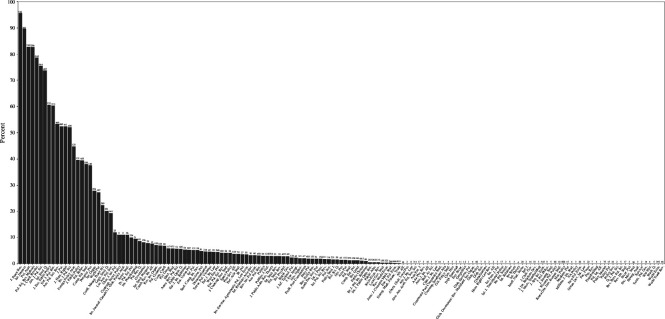
Open data by journal.

**Figure 9. F9:**
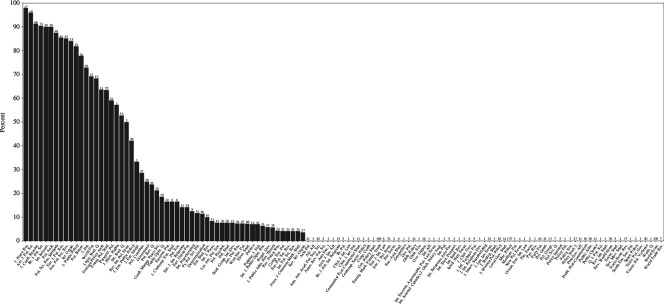
Open data by journal in 2020.

**Figure 10. F10:**
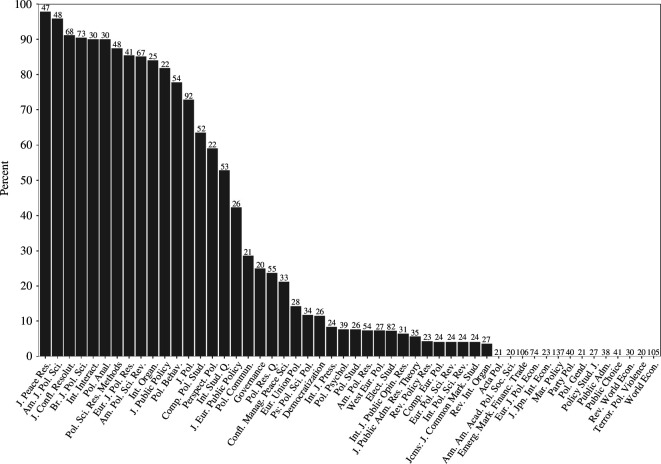
Open data by journal (with over 20 statistical inference papers) in 2020.

**Figure 12. F12:**
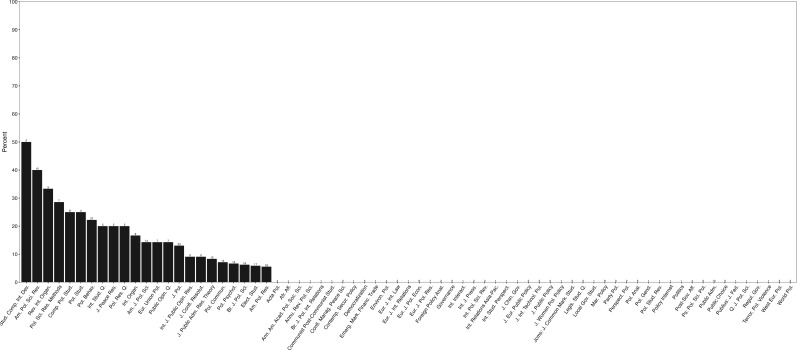
Preregistration by journal in 2020.

**Figure 13. F13:**
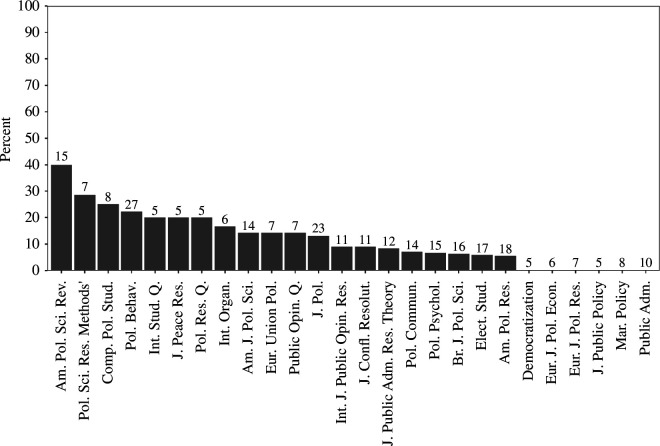
Preregistration by journal (with over five experimental papers) in 2020.

**Figure 14.**
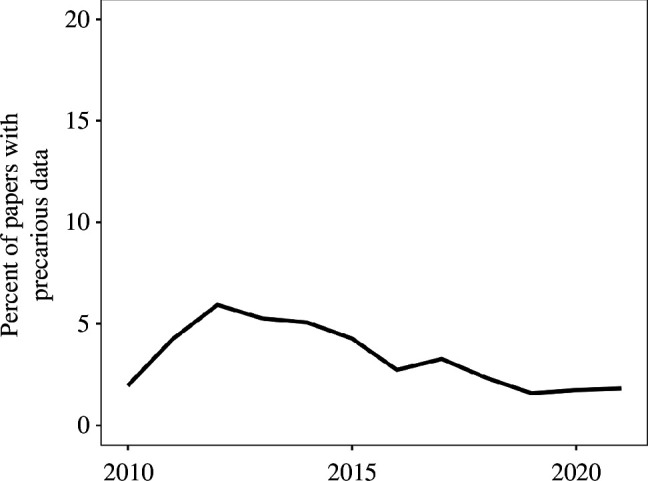
Precarious data by year.

## Data Availability

All code and non-copyright data necessary to computationally reproduce this paper may be found in its GitHub repository, preserved by Zenodo [59]. We used R Markdown (.Rmd) to write the manuscript. The master dataset, with each article as a row, is included, as well as the R scripts (.R files) used to generate all the figures and tables. The R scripts used to classify the full-text papers and link papers to the data repositories are also included. However, we cannot make >100 000 full-text articles publicly available.

## Footnotes

^1^
Key [9] analyses 586 articles in six top political science and international relations journals—some of which have already adopted compulsory data availability policies—for 2014 and 2015. Stockemer *et al.* [10] analyse data availability in the articles of three journals in 2015. Grossman & Pedahzur [11] analyse 92 articles published in the autumn 2019 issues of six journals and argue that the field is now approaching a ‘replicability utopia’. Rainey *et al.* [12] sample 6000 DOIs and use four human coders to identify 1413 that have quantitative data.

^2^
A complete list of the journals can be found in appendix A (table 1).

^3^
The three journals analysed were *Electoral Studies*, *Party Politics* and *Journal of Elections, Public Opinion and Parties*.

^4^
The six journals analysed were *American Political Science Review*, *American Journal of Political Science*, *British Journal of Political Science*, *International Organization*, *Journal of Politics* and *Political Analysis.*

^5^
Summaries of these debates can be found in Lupia & Elman [25] and on the Dialogue on DART website [26].

^6^
We discuss these issues further in §3.

^7^
For instance, Lenz & Sahn [34] find that over 30% of observational studies published in the *American Journal of Political Science* rely on suppression effects to achieve statistical significance. Being able to determine the influence of suppression effects requires access to the original data.

^8^
As some journals publish both political science and international relations articles, the top 100 journals in each category overlapped. These steps can be found in ./code/R_updated/1.1_pull-crossref-data.R.

^9^
A more complete discussion of the choice of this field is found in the ./rr_code/rr_crossref_date_field_choice_analysis.R file, in this article’s code repository, which also shows the unintentional omission of seven journals due to this decision. Subsequent references to code files will all be found in the repository.

^10^
These steps can be found in ./R_updated/2.1_gather-process-fulltext.R and ./code/R_updated/1.2_join-clean-crossref-data.R.

^11^
See ./code/R_original/count_quant_terms.R.

^12^
A custom function was preferable to existing text analysis libraries like quanteda because of our need to capture regular expressions and asterisks.

^13^
./code/R_updated/3.1_classify-fulltext-paper.R describes the classification process and points to the complete list of hundreds of terms used to identify statistical inference papers –./output/quant_dict_w_cats.txt.

^14^
As an additional robustness check to predict open data and statistical inference papers, we also attempted to use the Claude 3 Haiku model by Anthropic, but discontinued this experiment due to time and resource constraints, as detailed in the letter to reviewers in the replication materials. The code performing this work is in ./code/R_original/ml_classifier_quant_papers.R, ./code/R_updated/3.1_classifyfulltext-papers.R and ./rr_code/code/classify_quant_papers.py.

^15^
See ./code/R_updated/4.1a_dataverse_query_journals.R.

^16^
See ./code/R_updated/4.1_query-dataverse-with-titles.R.

^17^
See ./code/R_updated/4.1b_dataverse_link_to_papers.R.

^18^
See ./code/R_updated/4.1a_dataverse_query_journals.R.

^19^
Terms like ‘replication data’ are used in political science to refer to computational reproducibility materials such as open data and code.

^20^
See ./code/R_original/supplementary_replication_mentions.R and ./code/R_updated/4.4_precarious-data.R.

^21^
Because we identified only five articles that referred to such repositories where the article was not already coded as having open data, we did not include them in the results. See ./code/R_original/test_precarious_data.R.

^22^
See ./code/R_original/validate_dart_analysis.R.

^23^
See ./code/R_updated/5.2_identify-prereg.R, ./code/R_original/count_experimental_papers.R and ./code/R_original/count_prereg_papers.R.

^24^
./code/bash/rg_for_prereg_osf_egap_papers.sh.

^25^
Gary King illustrated that by 1988 almost half of publications in the *American Political Science Review* were quantitative.

^26^
The cut-off was established to focus on journals that publish more quantitative papers and for ease of viewing—the graph with all 158 journals with at least one statistical inference paper is very large and is located in appendix A.

^27^
The journals with over 50% open data are the *American Journal of Political Science*, the *American Political Science Review*, the *British Journal of Political Science*, *European Journal of Political Research*, *International Interactions*, *International Studies Quarterly*, *Journal of Conflict Resolution*, *Journal of Peace Research*, *Journal of Politics*, *Political Analysis* and *Political Science Research and Methods*. Those with over 20% open data include the aforementioned journals as well as *Comparative Political Studies*, *Conflict Management and Peace Science*, *International Organisation*, *Legislative Studies Quarterly* and *Political Behaviour*.

^28^
A total of 27 journals signed the DA-RT statement. The majority of these journals publish quantitative research (as can be seen in figure 2). Note that there are actually 20 DA-RT signatory journals in our dataset, but 4 of them have an insignificant number of statistical inference publications and so we omit them from the analysis.

^29^
Journals like the *Journal of Politics* require authors to disclose a preregistration report or justify why they did not preregister their experiment.

^30^
For experiments, we acknowledge that these are by no means definitive criteria on which to judge the trustworthiness of a paper or finding. These practices should accompany efforts to build confidence in a finding through direct and conceptual replications.

## Appendix A

### Identifying papers relying on data analysis

We defined data analysis papers as those that made any display or presentation of numerical data, most commonly in tables and graphs. Maps that included data-rich overlays and required software to produce were included in this category (table 1, figures 6 [Fig F7] [Fig F8] [Fig F9] [Fig F10] [Fig F11] [Fig F12] [Fig F13]–14).

**Table 1.** Journals analysed as ranked by Journal Citation Report 2020.

journal name	category 1	category 2	JIF 2020
POLITICAL ANALYSIS	PS		8.60
Annual Review of Political Science	PS		8.10
International Affairs	IR		7.90
Political Communication	PS		7.90
American Political Science Review	PS		7.80
Review of International Organizations	IR	PS	7.80
International Security	IR		7.50
Journal of European Public Policy	PS		7.30
Journal of Public Administration Research and Theory	PS		7.00
Environmental Politics	PS		6.70
International Journal of Press-Politics	PS		6.60
INTERNATIONAL ORGANIZATION	IR	PS	6.30
POLITICAL BEHAVIOR	PS		6.20
AMERICAN JOURNAL OF POLITICAL SCIENCE	PS		6.10
Regulation & Governance	PS		5.40
JOURNAL OF DEMOCRACY	PS		5.30
BRITISH JOURNAL OF POLITICAL SCIENCE	PS		5.20
COMPARATIVE POLITICAL STUDIES	PS		5.10
POLICY STUDIES JOURNAL	PS		5.10
EUROPEAN JOURNAL OF POLITICAL RESEARCH	PS		4.90
FOREIGN AFFAIRS	IR		4.80
NEW POLITICAL ECONOMY	IR	PS	4.70
REVIEW OF INTERNATIONAL POLITICAL ECONOMY	IR	PS	4.70
Socio-economic Review	PS		4.40
POLITICAL PSYCHOLOGY	PS		4.30
MARINE POLICY	IR		4.20
Policy and Society	PS		4.20
PUBLIC OPINION QUARTERLY	PS		4.20
European Political Science Review	PS		4.10
Geopolitics	PS		4.10
Global Environmental Politics	IR	PS	4.10
JOURNAL OF PEACE RESEARCH	IR	PS	4.10
EUROPEAN JOURNAL OF INTERNATIONAL RELATIONS	IR		4.00
Journal of Common Market Studies	IR	PS	4.00
WEST EUROPEAN POLITICS	PS		4.00
Territory Politics Governance	PS		3.90
GOVERNANCE—AN INTERNATIONAL JOURNAL OF POLICY ADMINISTRATION AND INSTITUTIONS	PS		3.80
Perspectives on Politics	PS		3.80
Policy and Internet	PS		3.80
POLICY AND POLITICS	PS		3.80
Political Science Research and Methods	PS		3.80
South European Society and Politics	PS		3.80
International Political Sociology	IR	PS	3.70
POLITICAL GEOGRAPHY	PS		3.70
PUBLIC ADMINISTRATION	PS		3.70
Chinese Journal of International Politics	IR		3.60
Cooperation and Conflict	IR	PS	3.60
JOURNAL OF CONFLICT RESOLUTION	IR	PS	3.50
JOURNAL OF POLITICS	PS		3.50
SECURITY DIALOGUE	IR		3.50
EUROPEAN UNION POLITICS	PS		3.40
WORLD POLITICS	IR	PS	3.40
COMMON MARKET LAW REVIEW	IR		3.30
GOVERNMENT AND OPPOSITION	PS		3.30
AFRICAN AFFAIRS	PS		3.20
Political Studies Review	PS		3.20
AMERICAN JOURNAL OF INTERNATIONAL LAW	IR		3.10
Cambridge Review of International Affairs	IR	PS	3.10
Democratization	PS		3.10
POLITICS & SOCIETY	PS		3.10
Research & Politics	PS		3.10
International Peacekeeping	IR		3.00
POST-SOVIET AFFAIRS	PS		3.00
INTERNATIONAL STUDIES QUARTERLY	IR	PS	2.90
MILLENNIUM—JOURNAL OF INTERNATIONAL STUDIES	IR		2.90
International Theory	IR	PS	2.80
PARTY POLITICS	PS		2.80
International Studies Review	IR	PS	2.70
LOCAL GOVERNMENT STUDIES	PS		2.70
POLITICAL SCIENCE QUARTERLY	PS		2.70
REVIEW OF INTERNATIONAL STUDIES	IR		2.70
TERRORISM AND POLITICAL VIOLENCE	IR	PS	2.70
CONFLICT MANAGEMENT AND PEACE SCIENCE	IR		2.60
Contemporary Security Policy	IR	PS	2.60
International Environmental Agreements—Politics Law and Economics	PS		2.60
Mediterranean Politics	IR	PS	2.60
POLITICAL RESEARCH QUARTERLY	PS		2.60
AMERICAN POLITICS RESEARCH	PS		2.50
British Politics	PS		2.50
International Studies Perspectives	IR		2.50
Journal of Public Policy	PS		2.50
Politics	IR	PS	2.50
PS—POLITICAL SCIENCE & POLITICS	PS		2.50
PUBLIUS—THE JOURNAL OF FEDERALISM	PS		2.50
SECURITY STUDIES	IR		2.50
Acta Politica	PS		2.40
British Journal of Politics & International Relations	IR	PS	2.40
European Journal of Political Economy	PS		2.40
JOURNAL OF STRATEGIC STUDIES	IR	PS	2.40
PACIFIC REVIEW	IR		2.40
POLITICAL STUDIES	PS		2.40
EMERGING MARKETS FINANCE AND TRADE	IR		2.30
International Relations of the Asia-Pacific	IR		2.30
Journal of Chinese Governance	PS		2.30
Social Movement Studies	PS		2.30
German Politics	PS		2.20
Globalizations	IR		2.20
Journal of Information Technology & Politics	PS		2.20
Journal of International Relations and Development	IR	PS	2.20
Journal of Intervention and Statebuilding	IR		2.20
JOURNAL OF POLITICAL PHILOSOPHY	PS		2.20
LEGISLATIVE STUDIES QUARTERLY	PS		2.20
STUDIES IN COMPARATIVE INTERNATIONAL DEVELOPMENT	IR	PS	2.20
ANNALS OF THE AMERICAN ACADEMY OF POLITICAL AND SOCIAL SCIENCE	PS		2.10
BULLETIN OF THE ATOMIC SCIENTISTS	IR		2.10
Critical Policy Studies	PS		2.10
ELECTORAL STUDIES	PS		2.10
EUROPE-ASIA STUDIES	PS		2.10
Global Policy	IR	PS	2.10
International Feminist Journal of Politics	PS		2.10
INTERNATIONAL JOURNAL OF PUBLIC OPINION RESEARCH	PS		2.10
International Relations	IR		2.10
Politics & Gender	PS		2.10
Politics and Governance	PS		2.10
PROBLEMS OF POST-COMMUNISM	PS		2.10
Comparative European Politics	PS		2.00
INTERNATIONAL POLITICAL SCIENCE REVIEW	PS		2.00
Journal of Women Politics & Policy	PS		2.00
NEW LEFT REVIEW	PS		2.00
PUBLIC CHOICE	PS		2.00
Quarterly Journal of Political Science	PS		2.00
Review of Policy Research	PS		2.00
European Security	IR		1.90
Asia Europe Journal	IR		1.80
Ethics & International Affairs	IR		1.80
European Journal of International Law	IR		1.80
Foreign Policy Analysis	IR		1.80
Peacebuilding	IR		1.80
SURVIVAL	IR		1.70
Human Rights Law Review	IR		1.60
International Journal of Transitional Justice	IR		1.60
JOURNAL OF THE JAPANESE AND INTERNATIONAL ECONOMIES	IR		1.60
World Trade Review	IR		1.60
Journal of European Integration	IR		1.50
OCEAN DEVELOPMENT AND INTERNATIONAL LAW	IR		1.50
REVIEW OF WORLD ECONOMICS	IR		1.50
WASHINGTON QUARTERLY	IR		1.50
AUSTRALIAN JOURNAL OF INTERNATIONAL AFFAIRS	IR		1.40
Chinese Journal of International Law	IR		1.40
INTERNATIONAL INTERACTIONS	IR		1.40
Journal of Contemporary European Studies	IR		1.40
STANFORD JOURNAL OF INTERNATIONAL LAW	IR		1.40
WORLD ECONOMY	IR		1.40
Contemporary Southeast Asia	IR		1.30
Intelligence and National Security	IR		1.30
LATIN AMERICAN POLITICS AND SOCIETY	IR		1.30
SPACE POLICY	IR		1.20
ALTERNATIVES	IR		1.10
COMMUNIST AND POST-COMMUNIST STUDIES	IR		1.10
Revista Brasileira de Politica Internacional	IR		1.10
JOURNAL OF WORLD TRADE	IR		1.00
GLOBAL GOVERNANCE	IR		0.90
International Politics	IR		0.90
Asian Perspective	IR		0.80
INTERNATIONAL JOURNAL	IR		0.80
CORNELL INTERNATIONAL LAW JOURNAL	IR		0.70
Journal of Human Rights	IR		0.70
Asian Journal of WTO & International Health Law and Policy	IR		0.60
International Journal of Conflict and Violence	IR		0.60
Pacific Focus	IR		0.60
WAR IN HISTORY	IR		0.60
COLUMBIA JOURNAL OF TRANSNATIONAL LAW	IR		0.50
Journal of Cold War Studies	IR		0.50
MIDDLE EAST POLICY	IR		0.50
CURRENT HISTORY	IR		0.40
KOREAN JOURNAL OF DEFENSE ANALYSIS	IR		0.40
Diplomacy & Statecraft	IR		0.30
INTERNASJONAL POLITIKK	IR		0.30
Korea Observer	IR		0.30
Uluslararasi Iliskiler-International Relations	IR		0.30
Asia-Pacific Review	IR		
Foro Internacional	IR		
Global Society	IR		
IPRI Journal	IR		
Revista UNISCI	IR		
Strategic Analysis	IR		
